# Clinical and Radiographic Evaluation of Nanohydroxyapatite Powder in Combination with Polylactic Acid/Polyglycolic Acid Copolymer as Bone Replacement Graft in the Surgical Treatment of Intrabony Periodontal Defects: A Retrospective Case Series Study

**DOI:** 10.3390/ma13020269

**Published:** 2020-01-07

**Authors:** Simone Verardi, Teresa Lombardi, Claudio Stacchi

**Affiliations:** 1 Department of Periodontics, University of Washington, Seattle, WA 98195, USA; simover@yahoo.it; 2 Department of Health Sciences, University of “Magna Græcia”, 88100 Catanzaro, Italy; drteresalombardi@libero.it; 3 Department of Medical, Surgical and Health Sciences, University of Trieste, 34129 Trieste, Italy

**Keywords:** periodontal surgery, intrabony defect, alloplastic graft, nanohydroxyapatite, periodontal regeneration

## Abstract

The aim of this retrospective case series was to evaluate the clinical efficacy of nanohydroxyapatite powder (NHA) in combination with polylactic acid/polyglycolic acid copolymer (PLGA) as a bone replacement graft in the surgical treatment of intrabony periodontal defects. Medical charts were screened following inclusion and exclusion criteria. Periodontal parameters and periapical radiographs taken before surgery and at 12-month follow-up were collected. Intra-group comparisons were performed using a two-tailed Wilcoxon signed-rank test. Twenty-five patients (13 males, 12 females, mean age 55.1 ± 10.5 years) were included in the final analysis. Mean probing depth (PD) and clinical attachment level (CAL) at baseline were 8.32 ± 1.41 mm and 9.96 ± 1.69 mm, respectively. Twelve months after surgery, mean PD was 4.04 ± 0.84 mm and CAL was 6.24 ± 1.71 mm. Both PD and CAL variations gave statistically significant results (*p* < 0.00001). The mean radiographic defect depth was 5.54 ± 1.55 mm and 1.48 ± 1.38 mm at baseline and at 12-month follow-up, respectively (*p* < 0.0001). This case series, with the limitations inherent in the study design, showed that the combination of NHA and PLGA, used as bone replacement graft in intrabony periodontal defects, may give significant improvements of periodontal parameters at 12-month follow-up.

## 1. Introduction

Periodontitis is a chronic inflammatory disease caused by infection of the supporting tissues around teeth. The infection begins with colonization and growth of a small group of predominantly Gram-negative anaerobic bacteria and spirochetes [1]. These bacteria, organized in biofilms with other commensal species, colonize the root surface, eliciting a chronic inflammation which leads to the progressive destruction of alveolar bone and collagen fibers of the periodontal ligament, and to the formation of periodontal pockets [2]. Genetics, environmental and behavioral factors influence disease development, the exposure of susceptible individuals to its initiation, and the speed of progression [3]. The inflammatory process is regulated by several biomolecular response mediators that may induce a quite disproportional reaction, leading to connective tissue damage [4,5,6,7,8].

Alveolar bone resorption is one of the most typical features of periodontitis and it is mainly due to the altered equilibrium between the activity of osteoclasts and osteoblasts [9]. The clinical diagnosis of periodontitis is generally based on the presence and extent of periodontal pockets, on clinical attachment loss, on the pattern and extent of alveolar bone loss, or on a combination of the above [10]. Periodontal vertical bony defects have been associated with an increased risk of disease progression and tooth loss [11]. Grafting of vertical bone defects is usually suggested when the intrabony component measures at least 3 mm in depth and is surrounded by two or three bony walls [12,13]. Autogenous bone has been the most popular material when periodontal grafts were initially attempted [14]. Later on, other materials have been tested in order to fill vertical bony defects in a predictable way: allografts (especially, demineralized freeze-dried bone), alloplasts and xenografts are now commonly used as bone substitutes in periodontal surgery [15,16,17]. Among alloplastic materials, synthetic porous hydroxyapatite has been used as an osteoconductive bone substitute in oral and maxillofacial surgery for about 30 years [18]. The behavior of nano-sized hydroxyapatite as a grafting material has been investigated in various clinical applications, such as sinus floor elevation, ridge augmentation, alveolar ridge preservation and periodontal surgery [19,20,21,22,23]. Synthetic nanocrystalline hydroxyapatite (NHA) is currently commercially available in several formulations: powder (nanometric scale particles), granules (macro-scale particles), blocks or pastes (NHA crystals suspended in water). An alternative approach is represented by the combination of NHA powder with an additive, in order to prevent agglomeration of the ceramics and to improve surgical handling. One of the most used additives is poly lactic-co-glycolic acid (PLGA), a biomaterial widely spread in various fields of medicine because of its biodegradability and biocompatibility.

The aim of the present retrospective case series study was to evaluate the clinical efficacy of the combination NHA/PLGA as a grafting material in the surgical treatment of intrabony periodontal defects.

## 2. Materials and Methods

Medical records and periodontal charts of all patients treated with the combination NHA/PLGA as a periodontal graft were screened for inclusion in the present retrospective case series study. Surgical treatment was performed by three experienced clinicians (SV, TL, CS) from September 2009 to October 2018. All procedures were performed in full compliance with the Declaration of Helsinki and subsequent revisions (Fortaleza 2013), for investigations with human subjects. Patients authorized the use of their data for research purposes.

Inclusion criteria were:

- adult patients (>18 years old) affected by chronic periodontal disease;
- completion of initial periodontal therapy (oral hygiene instructions, scaling and root planning under local anesthesia, re-evaluation 6–8 weeks later);
- good general health;
- full mouth plaque control record <30% before surgery [24];
- presence of at least one periodontal defect with two and/or three remaining walls with a minimum of 3 mm intrabony component and probing depth (PD) ≥6 mm;
- clear understanding of benefits and possible risks of the procedure and signed informed consent.

Patients were excluded if they were:

- heavy smokers (>10 cigarettes a day), pipe and/or cigar smokers;
- alcohol and/or drug abusers;
- medical conditions that could affect wound healing at the time of surgery;
- insufficient information found in medical records (e.g., lack of clinical measurements);
- patients who did not comply to scheduled recall visits after surgery.

### 2.1. Surgical Procedure

**Figure 1:**
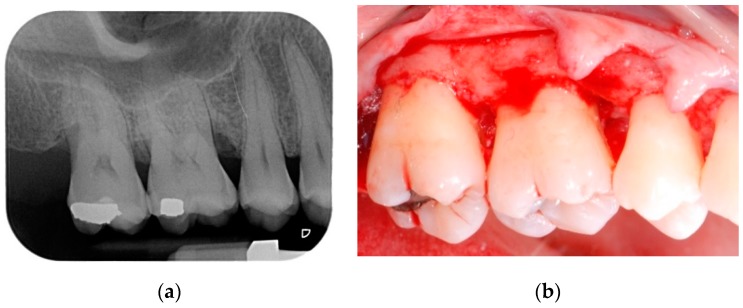
(**a**) Pre-operative periapical radiograph; (**b**) A full thickness flap was elevated in order to get access to the root and the periodontal intrabony defect.

**Figure 2:**
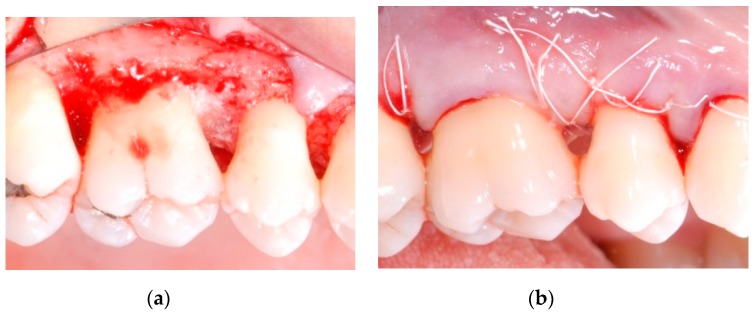
(**a**) After accurate debridement, the intrabony component of the defect was filled by a composite graft consisting in nanohydroxyapatite powder mixed with poly lactic-co-glycolic acid; (**b**) The buccal flap was then slightly released by longitudinal periosteal incision and closed with vertical mattress sutures.

All patients were treated under local anesthesia (articaine 4% with adrenaline 1:100,000). Incisions were performed with a 15b and/or 15c scalpel blade and papillae were preserved in the areas to be regenerated following a previously described technique [25]. Incisions were extended to the adjacent teeth in case multiple sites had to be treated. A full thickness flap was elevated in order to get access to the root and the periodontal intrabony defect (Figure 1a,b); a combination of ultrasonic device and manual curettes was used to debride the area and to remove calculus and inflammatory tissue. The intrabony component of the defect was then filled by a composite graft consisting of nanohydroxyapatite powder (NHA) with crystals varying in size between 70 and 100 nm (Neo Active Apatite, Ghimas, Casalecchio di Reno, Italy) mixed with poly lactic-co-glycolic acid (PLGA) (Fisiograft Gel, Ghimas, Casalecchio di Reno, Italy) (Figure 2a). The mix ratio was 1:1 by volume. The buccal flap was then slightly released by longitudinal periosteal incision and 5.0 monofilament sutures were used to obtain primary soft tissue closure with vertical mattress sutures (Figure 2b).

### 2.2. Post-Operative Care

**Figure 3:**
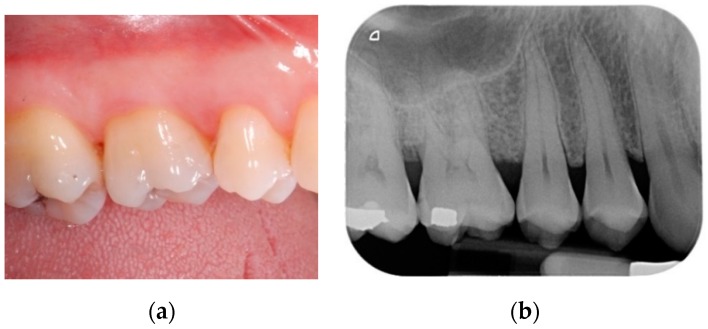
(**a**) Clinical view of the treated area at 12-month follow-up; (**b**) periapical radiograph at 12-month follow-up.

Patients were prescribed with doxyciclin 100 mg (2 tablets on the first day and then one tablet per day for 14 days) and ibuprofen 600 mg (2 tablets per day for 5 days). Three patients, who had previously reported recurrent gastritis, were also prescribed with pantoprazole 20 mg (one tablet every morning before breakfast for 10 days). Patients were instructed not to brush the treated area, but to rinse their mouth twice a day using a 0.20% chlorhexidine mouthwash, starting from the day after the surgery until suture removal. A cold diet and ice packs application were recommended for the first day after surgery. All patients were seen 7 days post-op; a second check was scheduled 2 weeks after the surgical procedure; at this time, the sutures were also removed. For the first 12 months after surgery, patients were scheduled for supportive periodontal therapy recalls every three months (Figure 3a,b).

### 2.3. Clinical Measurements

**Figure 4:**
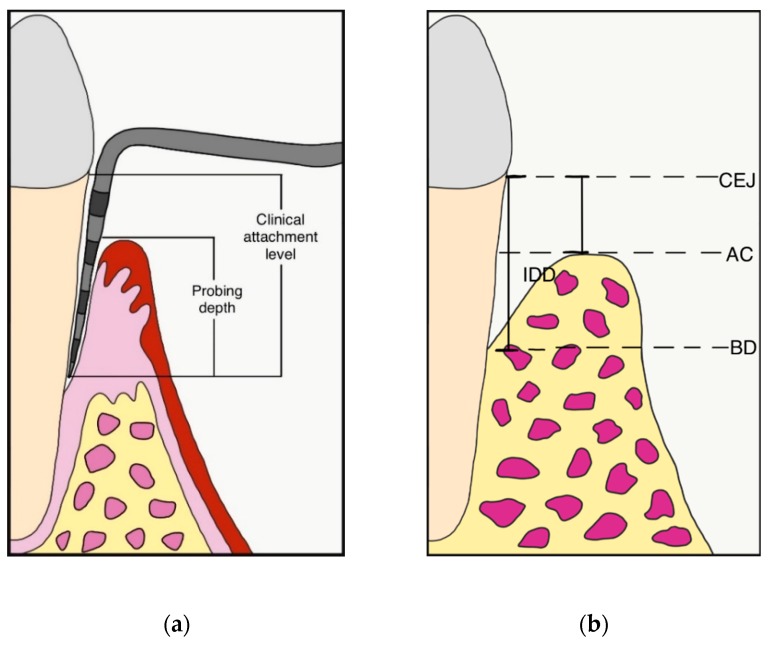
(**a**) Probing depth (PD) and clinical attachment level (CAL) measurement; (**b**) Intrabony defect depth (IDD) was defined as (CEJ-BD)—(CEJ-AC); CEJ: cement enamel junction; BD: bottom of the defect; AC: alveolar bone crest.

Periodontal parameters were collected at first visit, at re-evaluation after initial therapy, before surgery (baseline) and 12 months after the surgical procedure. The gingival index (GI) and plaque index (PI) were recorded [26], while probing depth (PD) and clinical attachment level (CAL) were measured at 6 sites per tooth (mesio-buccal, mid-buccal, disto-buccal, disto-lingual, mid-lingual, mesio-lingual) by using a periodontal probe (PCP-UNC 15, Hu-Friedy, Chicago, IL, USA) (Figure 4a). Measurements were rounded to the nearest millimeter. When the cemento-enamel junction (CEJ) could not be used for determining CAL, the restoration margin was used as a reference [27]. The site presenting the deepest PD was included in the final analysis.

All the clinical measurements were taken by three independent assessors, blinded to treatment assignment. Periapical digital radiographs were taken using a long-cone paralleling technique with a Rinn-type film holder, before surgery and at 12-month follow-up. The following linear measurements were taken by a single calibrated examiner, on a 30-inch led-backlit color diagnostic display, using measuring software (Image J 1.52a, National Institutes of Health, Bethesda, MD, USA): (1) distance from the CEJ to the most apical extension of the bony defect (CEJ-BD); (2) distance from the CEJ to the most coronal extension of the alveolar bone crest (CEJ-AC). The depth of the intrabony component of the defect (IDD) was defined as (CEJ-BD)—(CEJ-AC) (Figure 4b).

### 2.4. Statistical Analysis

Statistical analysis was performed by using Statistical Package Software for Social Sciences (SPSS for Windows, version 22.0, Chicago, IL, USA). The significance level was set at 0.05. The patient was defined as the statistical unit. A Kolmogorov–Smirnov test was performed to assess the normality distribution of continuous variables (GI, PI, CAL, PD, IDD). Intra-group comparisons (baseline/12 months) were performed using a two-tailed Wilcoxon signed-rank test.

## 3. Results

### 3.1. Study Population

Medical records of 37 patients were screened and 25 of them (13 males and 12 females), fulfilling inclusion criteria, were selected and evaluated in the final analysis. Mean age was 55.1 ± 10.5 years (range 44–79). Sixteen patients were non-smokers and nine patients were light smokers (<10 cigarettes/day). Patient demographics and characteristics are summarized in Table 1. Fourteen defects were localized in the lower arch and 11 defects in the upper arch.

### 3.2. Clinical Outcomes

Post-operative healing was uneventful in all patients. Statistical analysis failed to detect significant GI and PI changes from baseline to 12-month follow-up (*p* > 0.05). Mean PD and CAL at baseline were 8.32 ± 1.41 mm and 9.96 ± 1.69 mm, respectively. Twelve months after surgery, mean PD was 4.04 ± 0.84 mm and CAL was 6.24 ± 1.71 mm. Mean PD reduction was 4.28 ± 1.46 mm and mean CAL gain was 3.72 ± 1.17 mm. Both PD and CAL variations between baseline and 12-month follow-up yielded statistically significant results (*p* < 0.00001). Distribution of treated sites and clinical outcomes are listed in Table S1. Mean radiographic IDD was 5.54 ± 1.55 mm and 1.48 ± 1.38 mm at baseline and at 12-month follow-up, respectively (*p* < 0.0001). The mean radiographic defect fill was 4.06 ± 1.66 mm (73.3%).

## 4. Discussion

Bone replacement grafts represent a reliable surgical option in the treatment of periodontal intrabony defects, resulting in long-term improved probing depths and clinical attachment levels [28,29]. A recent systematic review showed that this treatment modality results in clinical outcomes generally similar to biological factors (enamel matrix derivative and recombinant human platelet-derived growth factor) [30]. Autologous bone and demineralized freeze-dried bone allograft have been the most widely used materials to graft intrabony defects for almost 30 years, demonstrating histological evidence of periodontal regeneration [31,32,33]. Synthetic bone substitutes, even if histological analyses demonstrated their limited regenerative potential [33], showed a satisfactory clinical behavior in numerous studies. The use of calcium sulfate [34], calcium phosphates [35], biphasic calcium composite [36] and various formulations of hydroxyapatite (HA) [37,38,39] allowed significant short- and long-term improvement of periodontal parameters. In periodontal surgery, HA has been used since the 1980s: Kenney et al. used a hydroxyapatite with a pore size of 190 to 220 microns in angular defects [18], reporting improvement of clinical parameters and evident bone formation at surgical re-entry. Subsequently, the same group described bone formation and lack of graft resorption in histological studies [40,41].

With the development of nanotechnology, nanoscale biomaterials have been studied to improve their biological properties. Nanostructured hydroxyapatite (NHA) can mimick the surface characteristics of the inorganic component of the native bone matrix, enhancing the regenerative performance. NHA favours the rapid formation of a stable microvasculature, an essential requisite to support the metabolic needs of bone-forming cells and newly formed tissue [42]. This complex process includes a biomolecular communication between endothelial cells, involved in the formation of the vascular network, macrophages and mesenchymal osteoprogenitor cells [43].

Mechanical, physicochemical and biological properties of the combination of NHA and PLGA have been widely investigated by previous preclinical studies, showing excellent biocompatibility, good osteoconductive activity, as well as good performance of host tissue response [44,45,46,47]. These properties paved the way to the use of this composite graft as a biocompatible scaffold for bone tissue engineering. In the present study, a 1:1 mix of NHA powder and PLGA gel was used as a bone replacement graft in the surgical treatment of intrabony defects. The relative proportion of the two materials was chosen in accordance with a previous in vitro study showing that the adhesion, proliferation, and osteogenic differentiation of bone marrow stromal stem cells with NHA/PLGA (50/50) were better than those with NHA/PLGA (20/80) [48]. Significant PD reduction (4.28 ± 1.46 mm) and CAL gain (3.72 ± 1.17 mm) were recorded one year after surgery: these outcomes are in accordance with a randomized controlled clinical trial by Kasaj et al. [20], comparing nanocrystalline hydroxyapatite paste (test) with open flap debridement (control) in intrabony defects treatment. At 6-month follow up, the test group showed significant PD reduction and CAL gain (3.9 ± 1.2 mm and 3.6 ± 1.6 mm, respectively), with values very similar to the ones recorded in the present study. The mean radiographic defect fill in the present study was 4.06 ± 1.66 mm, corresponding to a 73.3% fill of the mean baseline defect. This outcome is substantially in line with previous trials conducted with the use of alloplasts, with or without the addition of biological factors (platetet-rich fibrin, enamel matrix derivative) [49,50,51]. Furthermore, the clinical outcomes of the present study are in accordance with the results reported in trials using bone replacement grafts for the surgical treatment of periodontal infrabony defects included in a recent systematic review [30].

Some limitations of the present case series should be considered when interpreting the present results. First, the lack of a control group makes this study prone to selection bias. Further randomized controlled trials are necessary to confirm and generalize these preliminary findings. Second, due to the retrospective nature of the present investigation, operators collecting clinical parameters were not calibrated and radiographs were not standardized. Third, histological evaluation of the regenerated tissue is lacking: this additional analysis could give a more complete understanding of the features of the combination NHA/PLGA in periodontal regeneration.

## 5. Conclusions

This retrospective case series showed that the combination of NHA and PLGA, used as a bone replacement graft in intrabony periodontal defects, may give satisfactory results in terms of PD reduction, CAL gain and radiographic bone fill at 12-month follow-up. The limitations inherent in the study design make these results not generalizable to larger populations of patients. However, the present case series provides information that could generate hypotheses possibly leading to focused studies of a stronger design.

## Figures and Tables

**Table 1:** Patient demographics and characteristics at baseline.

Number	25
Gender	13 males (52%); 12 females (48%)
Age (mean ± SD; range)	55.1 ± 10.5 years; 44–79
Non smokers	16 (64%)
Light smokers *	9 (36%)

SD: standard deviation; * <10 cigarettes/day.

## Supplementary Materials

The following is available online at https://www.mdpi.com/1996-1944/13/2/269/s1, Table S1: Distribution of treated sites and clinical outcomes.

## References

[1] Haffajee A.D., Socransky S.S. (1994). Microbial etiological agents of destructive periodontal diseases. Periodontol. 2000.

[2] Page R.C., Offenbacher S., Schroeder H.E., Seymour G.J., Kornman K.S. (1997). Advances in the pathogenesis of periodontitis: Summary of developments, clinical implications and future directions. Periodontol. 2000.

[3] Könönen E., Gursoy M., Gursoy U.K. (2019). Periodontitis: A multifaceted disease of tooth-supporting tissues. J. Clin. Med..

[4] Loos B.G., Papantonopoulos G., Jepsen S., Laine M.L. (2015). What is the contribution of genetics to periodontal risk?. Dent. Clin. N. Am..

[5] Benakanakere M., Kinane D.F. (2012). Innate cellular responses to the periodontal biofilm. Front. Oral Biol..

[6] Cekici A., Kantarci A., Hasturk H., Van Dyke T.E. (2014). Inflammatory and immune pathways in the pathogenesis of periodontal disease. Periodontol. 2000.

[7] Damgaard C., Holmstrup P., Van Dyke T.E., Nielsen C.H. (2015). The complement system and its role in the pathogenesis of periodontitis: Current concepts. J. Periodontal Res..

[8] Verardi S., Page R.C., Ammons W.F., Bordin S. (2007). Differential chemokine response of fibroblast subtypes to complement C1q. J. Periodontal Res..

[9] Belibasakis G.N., Bostanci N. (2012). The RANKL-OPG system in clinical Periodontol. J. Clin. Periodontol..

[10] Page R.C., Eke P.I. (2007). Case definitions for use in population-based surveillance of periodontitis. J. Periodontol..

[11] Papapanou P.N., Wennström J.L. (1991). The angular bony defect as indicator of further alveolar bone loss. J. Clin. Periodontol..

[12] Laurell L., Gottlow J., Zybutz M., Persson R. (1998). Treatment of intrabony defects by different surgical procedures. A literature review. J. Periodontol..

[13] Verardi S., Schuler R., Janakievski J. (2008). Guidelines for the use of osseous resective surgery in conjunction with implant placement. Pract. Proced. Aesthet. Dent..

[14] Karring T., Nyman S., Gottlow J., Laurell L. (1993). Development of the biological concept of guided tissue regeneration—Animal and human studies. Periodontol. 2000.

[15] Robinson E. (1969). Osseous coagulum for bone induction. J. Periodontol..

[16] Schwartz Z., Mellonig J.T., Carnes D.L., de la Fontaine J., Cochran D.L., Dean D.D., Boyan B.D. (1996). Ability of commercial demineralized freeze-dried bone allograft to induce new bone formation. J. Periodontol..

[17] Schwartz Z., Weesner T., van Dijk S., Cochran D.L., Mellonig J.T., Lohmann C.H., Carnes D.L., Goldstein M., Dean D.D., Boyan B.D. (2000). Ability of deproteinized cancellous bovine bone to induce new bone formation. J. Periodontol..

[18] Kenney E.B., Lekovic V., Han T., Carranza F.A., Dimitrijevic B. (1985). The use of a porous hydroxylapatite implant in periodontal defects. I. Clinical results after six months. J. Periodontol..

[19] Turco G., Porrelli D., Marsich E., Vecchies F., Lombardi T., Stacchi C., Di Lenarda R. (2018). Three-dimensional bone substitutes for oral and maxillofacial surgery: Biological and structural characterization. J. Funct. Biomater..

[20] Kasaj A., Röhrig B., Zafiropoulos G.G., Willershausen B. (2008). Clinical evaluation of nanocrystalline hydroxyapatite paste in the treatment of human periodontal bony defects—A randomized controlled clinical trial: 6-month results. J. Periodontol..

[21] Canullo L., Sisti A. (2010). Early implant loading after vertical ridge augmentation (VRA) using e-PTFE titanium-reinforced membrane and nano-structured hydroxyapatite: 2-year prospective study. Eur. J. Oral Implantol..

[22] Stacchi C., Lombardi T., Oreglia F., Alberghini Maltoni A., Traini T. (2017). Histologic and histomorphometric comparison between sintered nanohydroxyapatite and anorganic bovine xenograft in maxillary sinus grafting: A split-mouth randomized controlled clinical trial. BioMed Res. Int..

[23] Lombardi T., Bernardello F., Berton F., Porrelli D., Rapani A., Camurri Piloni A., Fiorillo L., Di Lenarda R., Stacchi C. (2018). Efficacy of alveolar ridge preservation after maxillary molar extraction in reducing crestal bone resorption and sinus pneumatization: A multicenter prospective case-control study. BioMed Res. Int..

[24] O’Leary T.J., Drake R.B., Naylor J.E. (1972). The plaque control record. J. Periodontol..

[25] Cortellini P., Pini Prato G.P., Tonetti M.S. (1995). The modified papilla preservation technique. A new surgical approach for interproximal regenerative procedures. J. Periodontol..

[26] Löe H. (1967). The gingival index, the plaque index and the retention index system. J. Periodontol..

[27] Röllke L., Schacher B., Wohlfeil M., Kim T.S., Kaltschmitt J., Krieger J., Krigar D.M., Reitmeir P., Eickholz P. (2012). Regenerative therapy of infrabony defects with or without systemic doxycycline. A randomized placebo-controlled trial. J. Clin. Periodontol..

[28] Rosen P.S., Reynolds M.A., Bowers G.M. (2000). The treatment of intrabony defects with bone grafts. Periodontol. 2000.

[29] Reynolds M.A., Aichelmann-Reidy M.E., Branch-Mays G.L. (2010). Regeneration of periodontal tissue: Bone replacement grafts. Dent. Clin..

[30] Kao R.T., Nares S., Reynolds M.A. (2015). Periodontal regeneration—Intrabony defects: A systematic review from the AAP Regeneration Workshop. J. Periodontol..

[31] Zubery Y., Kozlovsky A., Tal H. (1993). Histologic assessment of a contiguous autogenous transplant in a human intrabony defect. A case report. J. Periodontol..

[32] Masters L.B., Mellonig J.T., Brunsvold M.A., Nummikoski P.V. (1996). A clinical evaluation of demineralized freeze-dried bone allograft in combination with tetracycline in the treatment of periodontal osseous defects. J. Periodontol..

[33] Sculean A., Nikolidakis D., Nikou G., Ivanovic A., Chapple I.L., Stavropoulos A. (2015). Biomaterials for promoting periodontal regeneration in human intrabony defects: A systematic review. Periodontol. 2000.

[34] Orsini M., Orsini G., Benlloch D., Aranda J.J., Sanz M. (2008). Long-term clinical results on the use of bone replacement grafts in the treatment of intrabony periodontal defects. Comparison of the use of autogenous bone graft plus calcium sulfate to autogenous bone graft covered with a bioabsorbable membrane. J. Periodontol..

[35] Maroo S., Murthy K.R. (2014). Treatment of periodontal intrabony defects using β-TCP alone or in combination with rhPDGF-BB: A randomized controlled clinical and radiographic study. Int. J. Periodontics Restor. Dent..

[36] Stein J.M., Fickl S., Yekta S.S., Hoischen U., Ocklenburg C., Smeets R. (2009). Clinical evaluation of a biphasic calcium composite grafting material in the treatment of human periodontal intrabony defects: A 12-month randomized controlled clinical trial. J. Periodontol..

[37] Galgut P.N., Waite I.M., Bookshaw J.D., Kingston C.P. (1992). A 4-year controlled clinical study into the use of a ceramic hydroxylapatite implant material for the treatment of periodontal bone defects. J. Clin. Periodontol..

[38] Yukna R.A., Krauser J.T., Callan D.P., Evans G.H., Cruz R., Martin M. (2002). Thirty-six month follow-up of 25 patients treated with combination anorganic bovine-derived hydroxyapatite matrix (ABM)/cell-binding peptide (P-15) bone replacement grafts in human infrabony defects. I. Clinical findings. J. Periodontol..

[39] Scabbia A., Trombelli L. (2004). A comparative study on theuse of a HA/collagen/chondroitin sulphate biomaterial (Biostite) and a bovine-derived HA xenograft (Bio-Oss) in the treatment of deep intra-osseous defects. J. Clin. Periodontol..

[40] Carranza F.A., Kenney E.B., Lekovic V., Talamante E., Valencia J., Dimitrijevic B. (1987). Histologic study of healing of human periodontal defects after placement of porous hydroxylapatite implants. J. Periodontol..

[41] Kenney E.B., Lekovic V., Sa Ferreira J.C., Han T., Dimitrijevic B., Carranza F.A. (1986). Bone formation within porous hydroxylapatite implants in human periodontal defects. J. Periodontol..

[42] Fang C.H., Lin Y.W., Lin F.H., Sun J.S., Chao Y.H., Lin H.Y., Chang Z.C. (2019). Biomimetic synthesis of nanocrystalline hydroxyapatite composites: Therapeutic potential and effects on bone regeneration. Int. J. Mol. Sci..

[43] Yang C., Zhao C., Wang X., Shi M., Zhu Y., Jing L., Wu C., Chang J. (2019). Stimulation of osteogenesis and angiogenesis by micro/nano hierarchical hydroxyapatite via macrophage immunomodulation. Nanoscale.

[44] Lock J., Nguyen T.Y., Liu H. (2012). Nanophase hydroxyapatite and poly(lactide-co-glycolide) composites promote human mesenchymal stem cell adhesion and osteogenic differentiation in vitro. J. Mater. Sci. Mater. Med..

[45] Qian J., Xu W., Yong X., Jin X., Zhang W. (2014). Fabrication and in vitro biocompatibility of biomorphic PLGA/nHA composite scaffolds for bone tissue engineering. Mater. Sci. Eng. C.

[46] Li D., Sun H., Jiang L., Zhang K., Liu W., Zhu Y., Fangteng J., Shi C., Zhao L., Sun H. (2014). Enhanced biocompatibility of PLGA nanofibers with gelatin/nano-hydroxyapatite bone biomimetics incorporation. ACS Appl. Mater. Interfaces.

[47] Holmes B., Bulusu K., Plesniak M., Zhang L.G. (2016). A synergistic approach to the design, fabrication and evaluation of 3D printed micro and nano featured scaffolds for vascularized bone tissue repair. Nanotechnology.

[48] He S., Lin K.F., Sun Z., Song Y., Zhao Y.N., Wang Z., Bi L., Liu J. (2016). Effects of nano-hydroxyapatite/Poly (DL-lactic-co-glycolic acid) microsphere-based composite scaffolds on repair of bone defects: Evaluating the role of nano-hydroxyapatite content. Artif. Organs.

[49] Liu Y., Hu B., Zhou J., Li W., Liu Q., Song J. (2017). The effect of enamel matrix derivative alone versus in combination with alloplastic materials to treat intrabony defects: A meta-analysis. Int. J. Periodontics Restor. Dent..

[50] Pradeep A.R., Bajaj P., Rao N.S., Agarwal E., Naik S.B. (2017). Platelet-rich fibrin combined with a porous hydroxyapatite graft for the treatment of 3-wall intrabony defects in chronic periodontitis: A randomized controlled clinical trial. J. Periodontol..

[51] Bodhare G.H., Kolte A.P., Kolte R.A., Shirke P.Y. (2019). Clinical and radiographic evaluation and comparison of bioactive bone alloplast morsels when used alone and in combination with platelet-rich fibrin in the treatment of periodontal intrabony defects—A randomized controlled trial. J. Periodontol..

