# Supplementary material for: Clinical and Radiographic Evaluation of Nanohydroxyapatite Powder in Combination with Polylactic Acid/Polyglycolic Acid Copolymer as Bone Replacement Graft in the Surgical Treatment of Intrabony Periodontal Defects: A Retrospective Case Series Study
Source: Materials (Basel). 2020 Jan 7;13(2):269. doi: 10.3390/ma13020269 (PMC7014298; doi:10.3390/ma13020269)
Supplement: Supplementary file 1 [file materials-13-00269-s001.pdf]

materials

MDPI

*Supplementary*

# Clinical and Radiographic Evaluation of Nanohydroxyapatite Powder in Combination with Polylactic Acid/Polyglycolic Acid Copolymer as Bone Replacement Graft in the Surgical Treatment of Intrabony Periodontal Defects: A Retrospective Case Series Study

**Simone Verardi [1], Teresa Lombardi [2] and Claudio Stacchi [3,*]**

**Table S1.** Distribution of treated sites and clinical outcomes.

| | | PD | | | CAL | | |
|---|---|---|---|---|---|---|---|
| Patient No. | Tooth | Baseline (mm) | 12-month (mm) | PD reduction (mm) | Baseline (mm) | 12-month (mm) | CAL gain (mm) |
| 1 | 43 | 11 | 4 | 7 | 12 | 7 | 5 |
| 2 | 32 | 9 | 5 | 4 | 10 | 7 | 3 |
| 3 | 44 | 7 | 4 | 3 | 9 | 7 | 2 |
| 4 | 31 | 9 | 4 | 5 | 12 | 10 | 2 |
| 5 | 11 | 12 | 4 | 8 | 14 | 11 | 3 |
| 6 | 12 | 8 | 2 | 6 | 10 | 5 | 5 |
| 7 | 11 | 8 | 4 | 4 | 10 | 7 | 3 |
| 8 | 21 | 11 | 4 | 7 | 13 | 8 | 5 |
| 9 | 46 | 8 | 4 | 4 | 11 | 7 | 4 |
| 10 | 44 | 8 | 3 | 5 | 9 | 4 | 5 |
| 11 | 36 | 9 | 4 | 5 | 11 | 5 | 6 |
| 12 | 46 | 8 | 5 | 3 | 10 | 6 | 4 |
| 13 | 35 | 7 | 3 | 4 | 8 | 4 | 4 |
| 14 | 14 | 7 | 3 | 4 | 9 | 6 | 3 |
| 15 | 15 | 7 | 4 | 3 | 8 | 5 | 3 |
| 16 | 35 | 7 | 4 | 3 | 11 | 6 | 5 |
| 17 | 15 | 8 | 4 | 4 | 8 | 6 | 2 |
| 18 | 26 | 10 | 6 | 4 | 12 | 7 | 5 |
| 19 | 46 | 8 | 5 | 3 | 9 | 6 | 3 |
| 20 | 16 | 9 | 4 | 5 | 10 | 5 | 5 |
| 21 | 36 | 8 | 4 | 4 | 9 | 6 | 3 |
| 22 | 43 | 8 | 5 | 3 | 10 | 7 | 3 |
| 23 | 15 | 7 | 5 | 2 | 8 | 6 | 2 |
| 24 | 43 | 7 | 4 | 3 | 8 | 4 | 4 |
| 25 | 16 | 7 | 3 | 4 | 8 | 4 | 4 |
| **Mean** | | 8.32 | 4.04 | 4.28 | 9.96 | 6.24 | 3.72 |
| **SD (±)** | | 1.41 | 0.84 | 1.46 | 1.69 | 1.71 | 1.17 |

PD: probing depth; CAL: clinical attachment level; No.: number; mm: millimeters; SD: standard deviation.

© 2020 by the authors. Submitted for possible open access publication under the terms and conditions of the Creative Commons Attribution (CC BY) license (http://creativecommons.org/licenses/by/4.0/).
